# Verotoxigenic *Escherichia coli *O157:H7 from Swedish cattle; isolates from prevalence studies versus strains linked to human infections - A retrospective study

**DOI:** 10.1186/1746-6148-6-7

**Published:** 2010-01-29

**Authors:** Anna Aspán, Erik Eriksson

**Affiliations:** 1 Department of Bacteriology, National Veterinary Institute, SE-751 89 Uppsala, Sweden

## Abstract

**Background:**

Several cases of human infection caused by verotoxin-producing *Escherichia coli *(VTEC) O157:H7 in Sweden have been connected with cattle farm visits. Between 1996 and 2002, 18 farms were classified as the source of human cases with isolation of EHEC (Enterohaemorrhagic *Escherichia coli*) after VTEC O157:H7 had been isolated from cattle on those farms.

**Results:**

Characterization by phage typing and molecular methods of the strains isolated from these 18 farms, including PCR for virulence genes (*vtx*_1_, *vtx*_2 _and variants thereof, *eaeA *and EHEC-*hlyA*) and Pulsed-Field Gel Electrophoresis (PFGE), demonstrated a cluster of very similar strains from 16 farms. All were of phage type 4, carried the genes encoding the verotoxins VT2 and VT2c, intimin, EHEC-haemolysin and flagellin H7 as shown by PCR, and had identical or very similar PFGE patterns. When analysing strains in a prevalence study of VTEC O157:H7 from cattle at slaughter as well as from an on-farm prevalence study of dairy cattle, using the same typing methods, a rather wide variation was observed among the isolated VTEC O157:H7 strains.

**Conclusions:**

In Sweden, a limited group of genetically similar and highly pathogenic VTEC O157:H7 strains seem to predominate in direct or indirect transmission from cattle to man.

## Background

Verotoxin-producing *Escherichia coli *(VTEC) O157:H7 was first recognized as a human pathogen in 1982 [1]. As it was associated with consumption of undercooked 'hamburgers', it became known as 'the hamburger bug'. As it has subsequently been found that healthy cattle can harbour the bacterium, ruminants are now regarded as its main reservoir, though VTEC O157:H7 has been isolated from other animal species such as sheep, pigs, geese, gulls and pet animals [2]. Especially undercooked meat of bovine origin but also unpasteurized milk and other dairy products have been implicated in transmitting VTEC O157:H7 to humans. Another route for acquiring the infection is direct transmission from cattle, especially calves, for instance on 'open farms' where groups of children are welcome to visit. As the bacterium survives well in the environment, drinking water, vegetables irrigated with contaminated water, and public outdoor swimming pools have been mentioned as sources of community outbreaks. Direct person-to-person transmission also occurs, as the infectious dose of the bacterium is very low (reviewed by Gyles [2]).

Before 1995, very few human cases of enterohaemorrhagic *Escherichia coli *O157 infections were reported in Sweden, viz. between one and five cases annually. In 1995 at least two outbreaks caused by VTEC O157:H7 were identified, one in the autumn and one at Christmas time [3]. Between 1997 and 2002 the annual number of reported human VTEC O157 cases varied between 69 and 143 (annual incidence per 100,000 inhabitants between 0.7 and 1.6) of which approx. 60-80% were domestic cases http://www.smittskyddsinstitutet.se.

In the spring of 1996, one case of gastroenteritis in a child caused by VTEC O157:H7, was attributed to her visiting a cattle farm in southwest Sweden. At a follow-up inspection of cattle on this farm it was found that about half of the sampled animals were shedding VTEC O157:H7. Verotoxin type, phage type and the Pulsed-Field Gel Electrophoresis (PFGE) pattern of the isolates from cattle proved identical with the strain isolated from the child. Since then, cattle have been the primary suspect source of both direct and indirect human infection in Sweden.

To understand transmission patterns and detect outbreaks of VTEC O157:H7, subtyping methods such as phage typing and PFGE have commonly been used [4-8]. Further characterization of VTEC strains can be effected by detecting various virulence factors such as the verotoxins (VT) and by subtyping of the VT2 gene. Verotoxins are related to the Shiga toxins of *Shigella dysenteriae*, and there are two major *E. coli *verotoxins, VT1 and VT2. VT1 is a relatively uniform family of toxins [9], whereas the VT2 family is more diverse, comprising the variants VT2, VT2b, VT2c, VT2d, VT2e, VT2f and VT2g [10]. The VT2 genotype of a strain apparently influences its pathogenic ability and the variant *vtx*_2 _has been found to be significantly more common in strains isolated from patients who had developed haemolytic uraemic syndrome [11-14] and haemorrhagic colitis [11,15,16], than did the other *vtx *gene variants.

The purpose of this study was to compare phage types, verotoxin profiles and PFGE patterns of VTEC O157:H7 strains from cattle herds linked to human clinical cases, versus VTEC O157:H7 strains from other Swedish cattle. Another objective was to chart the geographical distribution of different VTEC O157:H7 variants in Sweden.

## Methods

### Strains isolated from Swedish cattle farms positive for VTEC O157: H7 implicated in transmitting disease to humans

In Sweden, if a County Medical Officer suspects that an infection by VTEC O157:H7 has been contracted by animal contact, he will inform the County Veterinary Officer, and immediately present a request to the Swedish Board of Agriculture to sample animals on the farm in question. Between 1996 and 2002, 37 farms were suspected of direct or indirect transmission of VTEC O157:H7 from cattle to humans. Each farm was initially sampled on one occasion. The number of faecal samples collected varied among the farms, but usually up to 100 samples were collected, with the sampling concentrated on young stock, as they are considered more likely to harbour the bacterium. Individual faecal samples were collected from rectum, wearing disposable rectal gloves, and were analysed either as individual samples or as pooled samples (five and five). If it was difficult to obtain individual faecal samples they could be replaced by composite pat samples (faeces collected from 5-10 picking sites from the box floor).

Of the 37 farms investigated, 18 were regarded as the source of infection for infected humans [17] who had been in contact with a presumed contaminated environment or food products. Also, the sampled cattle yielded VTEC O157:H7 strains having the same verotoxin composition and matching PFGE-banding patterns as the available human isolates. As it has previously been found that isolates with slight variations between PFGE banding patterns can originate from animals on the same farm [18], up to five cattle isolates, if available, were typed and compared with the human isolate(s). Also, some variation in PFGE banding patterns (up to two bands difference) could be allowed between the human and animals strains.

### Strains isolated from prevalence studies on slaughterhouses in Sweden

A slaughterhouse prevalence study to detect VTEC O157:H7 in Swedish cattle was performed between April 1996 and August 1997 [19]. The abattoirs included were geographically distributed throughout Sweden and together they accounted for more than 90% of all cattle slaughtered. Both dairy and beef cattle of all ages were sampled and the number of collected samples from each abattoir was proportional to the number of animals slaughtered. This study was then continued with a similar design, from 1998 to 2002. These studies were a part of a national monitoring programme intended to establish baseline data on prevalence of VTEC O157:H7 in cattle at slaughter and to determine the geographical distribution of VTEC O157:H7 in the Swedish cattle population. During the first prevalence study (1996-97) 37 bovine isolates of VTEC O157:H7 were found, and in the following studies (1998-2002) 111 isolates were collected.

### Isolates from faecal sampling on dairy cattle farms

During 1998-2000, in a study performed by Eriksson and colleagues [20], 371 farms were visited and altogether 7,397 individual faecal samples were collected. The farms were randomly selected from all over Sweden. 84 VTEC O157:H7 isolates were collected from 33 different dairy farms.

### Typing of strains

All bovine VTEC O157:H7 isolates from the slaughterhouse samplings (*n *= 37 +111) and all isolates from the dairy farm study (*n *= 84) were typed for virulence genes and subtyped by PFGE. From the 18 farms associated with human infections up to five isolates per farm were typed accordingly.

Virulence typing was performed by PCR to identify genes coding for VT1 and VT2 (*vtx*_1 _and *vtx*_2_), intimin (*eaeA*) and EHEC-haemolysin (EHEC-*hlyA) *according to Paton & Paton [21] and H7 (*fliC*) according to Gannon and colleagues [22]. VT2 -positive isolates were further typed to determine the VT2 gene variant, as described by Pierard and colleagues [23]. The following strains were included in each PCR analysis as positive and negative controls: Culture Collection, University of Gothenburg (CCUG) 42744 (*E. coli *O157 *vtx*_1 _^+ ^*vtx*_2 _^+ ^*eaeA*^+ ^EHEC-*hlyA*^+ ^*fliC*^+^) and CCUG 42901 (*E. coli *O157 lacking *vtx*_1_, *vtx*_2_, *eaeA*, EHEC-*hlyA*, and *fliC*).

PFGE was performed as described by Albihn and colleagues [19].

All isolates from the slaughterhouse samplings (*n *= 37+111) and selected representative strains from the dairy farm study (*n *= 33) and farms associated with human infections (*n *= 19) were phage typed. Principle for selection: if more than one PFGE pattern was found among strains isolated from a single farm, one representative of each pattern was phage typed. Phage typing using published methods was performed at the Laboratory of Enteric Pathogens (Central Public Health Laboratory, London, England) [24,25].

### Geographical distribution

In the slaughterhouse prevalence studies, geographical localization was noted for each VTEC O157:H7 positive animal as the postal code for the farm of origin. For a short period during 1999-2000, geographical information regarding the positive samples (*n *= 11) in the slaughterhouse studies was not obtained. Disregarding these 11 samples, the trace back farms and also the farms in the dairy cattle study have been denoted by their postal code. As tracing was not pursued further than to postal codes, the number of farms where positive samples were obtained, and their exact localization, could not be determined.

## Results

### Isolates from Swedish farms with cattle positive for VTEC O157:H7 implicated in transmitting disease to humans

**Figure 1:**
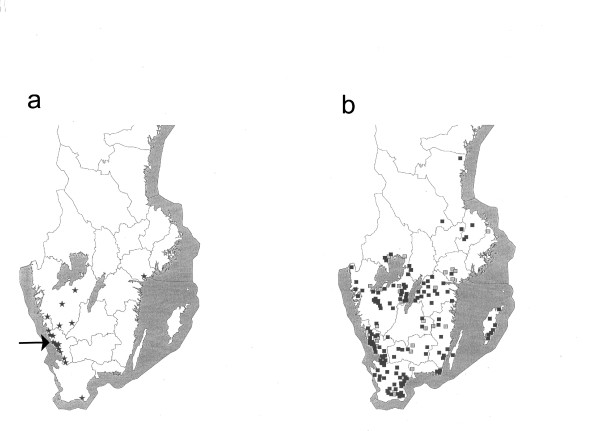
**Localization of cattle farms implicated in human cases versus origin of randomly isolated VTEC O157 strains**. Localization of cattle farms implicated in human EHEC cases viz-á-vis a map illustrating the origin of the randomly isolated VTEC O157 strains. (a) farms implicated in EHEC cases (*); (b) prevalence studies based on slaughterhouse sampling (■), prevalence study of on-farm dairy cattle (□). Arrow in (a) indicates the county of Halland.

In all 18 cases where VTEC O157:H7 could be isolated from bovine faecal samples collected on the farm implicated in transmitting disease to humans, the isolates were analysed further (Table 1, Figure 1a). Up to five VTEC O157:H7 isolates per farm, if available, were subjected to further typing. All isolates carried the EHEC-*hlyA*, *eaeA *and *fliC *genes. On each farm, all the analysed strains showed the same PFGE pattern, with one exception (farm No. 5 in Table 1).

**Table 1:** Cattle farms implicated in transmission of VTEC O157 from cattle to humans in Sweden between 1996 and 2002

Farm no	Year	County	Presence of *vtx *genes			Phage type	Type of farm	Connected cases
						
			***vtx***_**1**_	***vtx***_**2**_	***vtx***_**2*****c***_			
1	1996	Halland		*vtx*_2_	*vtx*_2*c*_	4	Dairy farm	1 child
2	1997	Halland		*vtx*_2_	*vtx*_2*c*_	4	Dairy farm including fattening pigs	1 child
3	1997	Halland		*vtx*_2_	*vtx*_2*c*_	4	Visiting farm, petting zoo	3 children
4	1997	Sörmland		*vtx*_2_	*vtx*_2*c*_	4	Dairy farm	1 child
5	1997	Älvsborg	*vtx*_1_		*vtx*_2*c*_	20/RDNC	Beef-producing farm	1 adult
6	1998	Halland		*vtx*_2_	*vtx*_2*c*_	4	Beef-producing farm	1 child
7	1998	Skaraborg		*vtx*_2_	*vtx*_2*c*_	4	Dairy farm	1 child
8	1998	Älvsborg		*vtx*_2_	*vtx*_2*c*_	2	Beef-producing farm	1 child
9	1999	Bohuslän		*vtx*_2_	*vtx*_2*c*_	4	Beef-producing farm	1 child*
10	1999	Halland		*vtx*_2_	*vtx*_2*c*_	4	Beef- producing farm including piglet production	1 child
11	1999	Halland		*vtx*_2_	*vtx*_2*c*_	4	Dairy farm	1 adult
12	2001	Halland		*vtx*_2_	*vtx*_2*c*_	4	Dairy farm	1 adult
13	2001	Skåne		*vtx*_2_	*vtx*_2*c*_	4	Dairy farm	1 child
14	2001	Halland		*vtx*_2_	*vtx*_2*c*_	4	Dairy farm	1 child
15	2001	Halland		*vtx*_2_	*vtx*_2*c*_	4	Dairy farm	1 child, 1 adult
16	2002	Halland		*vtx*_2_	*vtx*_2*c*_	4	Beef-producing farm including fattening pigs	1 child
17	2002	V-götland		*vtx*_2_	*vtx*_2*c*_	4	Dairy farm	1 child
18	2002	Halland		*vtx*_2_	*vtx*_2*c*_	4	Dairy farm	1 child

* Faecal sample from the child PCR-positive for v*tx*_2 _but no VTEC strain could be isolated from this patient

**Figure 2:**
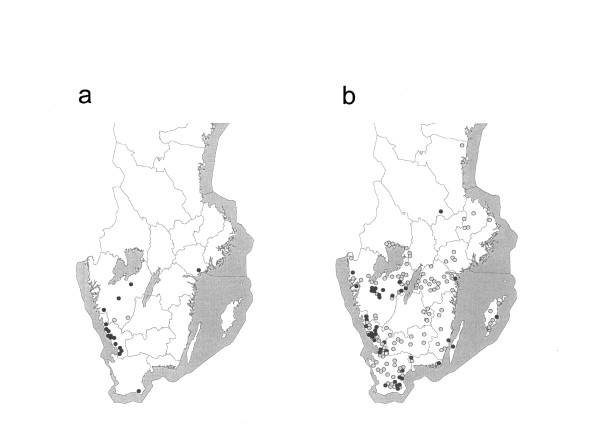
**Origin of VTEC O157 strains being PT4, *vtx*_2 _and *vtx*_2*c*_**. Map of (a) cattle farms implicated in human EHEC cases, viz-á-vis a map illustrating the geographical origin of the (b) randomly isolated VTEC O157 strains. Strains being PT4, *vtx*_2 _and *vtx*_2*c *_marked (●); other (○)

From 16 of 18 farms the VTEC O157:H7 isolates were of phage type 4, and carried both *vtx*_2 _and *vtx*_2*c *_genes (Table 1, Figure 2a). Two farms did not have isolates of PT4 (farms 5 and 8 in Table 1). On one of these farms (farm 8), strains of phage type 2 that carrying the *vtx*_2 _and *vtx*_2*c *_genes were isolated. However, in this investigation the human index case had a strain of PT4;*vtx*_2_, *vtx*_2*c*_, while in the sampling of asymptomatic relatives and friends, several cases of PT2;*vtx*_2_;*vtx*_2*c *_were found; i.e. this farm was regarded as implicated in human infection. The strains from farm No. 5 carried *vtx*_1 _and the *vtx*_2*c *_genes; those isolates belonged to phage type 20 and RDNC (= reacted but did not conform).

PFGE of the isolates revealed that on 16 of the 18 farms, all of which had strains from cattle belonging to PT4, the PFGE patterns clustered closely together and belonged either to a pattern which was common to eight of the farms, or to patterns differing by one to three bands from the predominant PFGE pattern. The PFGE patterns of the PT2 and the PT20/RDNC isolates from the other two farms differed by more than ten bands, when compared with each other and vis-à-vis the PT4 isolates.

### Isolates from the slaughterhouse prevalence study

During the first 18 months of the slaughterhouse prevalence study (1996-97) 37 VTEC O157:H7 positive samples were found. The isolates were divided among five known phage types; furthermore one single isolate and two isolates of previously undescribed phage types were found (Table 2) [19]. Phage types could be further divided by PFGE. All isolates possessed EHEC-*hlyA*, *eaeA *and *fliC *genes [19]; verotoxin variants are shown in Table 2. Subsequently, from 1998 to 2002, 111 strains of VTEC O157:H7 were isolated. Ten different phage types were present among the strains, as well as some phage types not previously described (RDNC) (Table 2), and PFGE could further divided within the phage types. All 111 isolates possessed EHEC-*hlyA*, *eaeA *and *fliC *genes; verotoxin genotypes are shown in Table 2. Six (16%) of 37 isolates from the study performed during 1996-97 and 34 (31%) of 111 isolates from 1998-2002 were of PT4;*vtx*_2_, *vtx*_2*c*_.

**Table 2:** Characteristics of strains of VTEC O157 isolated in the prevalence studies of cattle faeces at slaughterhouses in Sweden between 1996 and 2002

Presence of *vtx *genes			Phage type	No. of isolates	
		
***vtx***_1_	***vtx***_2_	***vtx***_2*c*_		1996/97	1998-2002
*vtx*_1_			8	1	1
	*vtx*_2_		4	2	2
		*vtx*_2*c*_	2		1
		*vtx*_2*c*_	8		1
		*vtx*_2*c*_	14	8	16
		*vtx*_2*c*_	32	2	2
		*vtx*_2*c*_	34		4
		*vtx*_2*c*_	49		2
		*vtx*_2*c*_	54		1
		*vtx*_2*c*_	RDNC	2	
	*vtx*_2_	*vtx*_2*c*_	1		1
	*vtx*_2_	*vtx*_2*c*_	2	1	2
	*vtx*_2_	*vtx*_2*c*_	4	6	34
	*vtx*_2_	*vtx*_2*c*_	24		1
*vtx*_1_		*vtx*_2*c*_	8	13	29
*vtx*_1_		*vtx*_2*c*_	14	1	1
*vtx*_1_		*vtx*_2*c*_	RDNC 1	1	1
*vtx*_1_		*vtx*_2*c*_	RDNC 2		10
*vtx*_1_		*vtx*_2*c*_	RDNC 3		1
*vtx*_1_		*vtx*_2*c*_	RDNC 4		1

**Total No. of isolates**				**37**	**111**

### Isolates from the on-farm prevalence study of dairy cattle

84 strains of VTEC O157:H7 were isolated from 33 different farms in the prevalence study of dairy cattle. When more than one strain was isolated from samples collected at the same farm, the PFGE patterns were either very similar, i.e. differing by only one or two bands, or indistinguishable. Phage types 2, 4, 8 and 14 were found, while three strains belonged to phage types not previously described (Table 3). All isolates carried EHEC-*hlyA*, *eaeA *and *fliC *genes; the verotoxin genotypes are shown in Table 3. VTEC O157:H7(PT4;*vtx*_2_, *vtx*_2*c*_) was found on seven (21%) of the 33 farms.

**Table 3:** Characteristics of strains of VTEC O157 isolated in the on-farm study of cattle faeces from dairy farms in Sweden between 1998 and 2000

Year of isolation	Strain code	County	Phage type	Presence of *vtx *genes		
				
				***vtx***_**1**_	***vtx***_**2**_	***vtx***_**2*****c***_
						
1998	PN 25	Sörmland	PT 4		*vtx*_2_	*vtx*_2*c*_
1998	PN 167	Kronoberg	PT 14			*vtx*_2*c*_
1998	PN 188	Skåne	PT 8	*Vtx*_1_		*vtx*_2*c*_
1998	PN 230	Halland	PT 4		*vtx*_2_	*vtx*_2*c*_
1998	PN 405	Jönköping	PT 8	*Vtx*_1_		*vtx*_2*c*_
1998	PN 431	Uppland	PT 14	*vtx*_1_		*vtx*_2*c*_
1998	PN 492	Värmland	PT 14			*vtx*_2*c*_
1999	PN 500	Sörmland	PT 8	*vtx*_1_		*vtx*_2*c*_
1999	PN 559	Sörmland	PT 8	*vtx*_1_		*vtx*_2*c*_
1999	PN 566	Skåne	PT 8	*vtx*_1_		*vtx*_2*c*_
1999	PN 573	Sörmland	PT 8	*vtx*_1_		*vtx*_2*c*_
1999	PN 585	Halland	PT 14			*vtx*_2*c*_
1999	PN 588	Halland	PT 4		*vtx*_2_	*vtx*_2*c*_
1999	PN 643	Kalmar	RDNC	*vtx*_1_		*vtx*_2*c*_
1999	PN 715	Värmland	PT 8	*vtx*_1_		*vtx*_2*c*_
1999	PN 746	Kalmar	RDNC	*vtx*_1_		*vtx*_2*c*_
1999	PN 781	Jönköping	PT 14			*vtx*_2*c*_
1999	PN 872	Skara	PT 14			*vtx*_2*c*_
1999	PN 899	Halland	PT 4		*vtx*_2_	*vtx*_2*c*_
1999	PN 905	Sörmland	PT 8	*vtx*_1_		*vtx*_2*c*_
1999	PN 931	Halland	PT 4		*vtx*_2_	*vtx*_2*c*_
1999	PN 1065	Skara	PT 8	*vtx*_1_		*vtx*_2*c*_
1999	PN 1095	Skara	PT 8	*vtx*_1_		*vtx*_2*c*_
1999	PN 1110	BKH	PT 14			*vtx*_2*c*_
2000	PN 1141	Skara	PT 4		*vtx*_2_	*vtx*_2*c*_
2000	PN 1167	Skåne	PT 8	*vtx*_1_		*vtx*_2*c*_
2000	PN 1217	Skara	PT 8	*vtx*_1_		*vtx*_2*c*_
2000	PN 1260	Skara	PT 8	*vtx*_1_		*vtx*_2*c*_
2000	PN 1328	Värmland	PT 8	*vtx*_1_		*vtx*_2*c*_
2000	PN 1376	Gotland	RDNC	*vtx*_1_		*vtx*_2*c*_
2000	PN 1394	Halland	PT 4		*vtx*_2_	*vtx*_2*c*_
2000	PN 1400	Halland	PT 2		*vtx*_2_	*vtx*_2*c*_
2000	PN 1599	Skara	PT 8	*vtx*_1_		*vtx*_2*c*_

### Geographical distribution

The geographical distribution of the 18 cattle farms implicated in transmission of disease to humans, is shown in Figure 1a. Most of the farms are in south-west Sweden, except for one farm on the east coast and one in the far south. VTEC O157:H7 (PT4;*vtx*_2_, *vtx*_2*c*_) was isolated from 16 of these farms. Eleven (68%) farms were situated in the County of Halland in southwest Sweden, see Figure 1.

**Figure 3:**
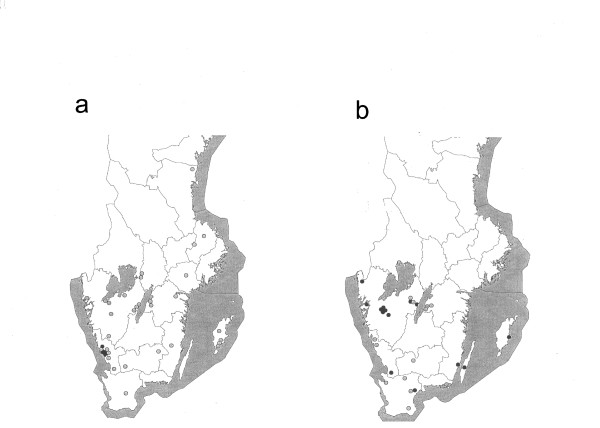
**Origin of randomly isolated VTEC O157:H7 strainsbeing PT4, *vtx*_2 _and *vtx*_2*c *_from two different time periods**. Map (a) illustrating the geographical origin of the 37 VTEC O157 isolates from the slaughterhouse survey performed between 1996-97, viz-à-viz a map (b) illustrating the geographical origin of the 29 VTEC O157 strains isolated in the 2002 slaughterhouse prevalence study. Strains being PT4, *vtx*_2 _and *vtx*_2*c *_marked (●); other (○).

The geographical distribution of the strains isolated in the slaughterhouse study and in the on-farm dairy cattle prevalence study is shown in Figure 1b. Strains of VTEC O157:H7 (PT4;*vtx*_2_, *vtx*_2*c*_) were more commonly found in Halland (Figure 2b). In the first slaughterhouse study performed in 1996-97, all the six VTEC O157:H7 (PT4;*vtx*_2_, *vtx*_2*c*_) strains isolated (altogether 37 strains), originated from animals in a limited part of Halland (Figure 3a). In the prevalence study on dairy cattle farms, VTEC O157:H7 (PT4;*vtx*_2_, *vtx*_2*c*_) was isolated on seven farms, of which five (71%) were situated in Halland. Thus, except for one dairy farm, all instances of VTEC O157:H7 (PT4;*vtx*_2_, *vtx*_2*c*_) isolated before 1998 were from cattle sampled in Halland.

After 1997, VTEC O157:H7 (PT4;*vtx*_2_, *vtx*_2*c*_) was isolated more frequently from cattle in other parts of Sweden (Figure 2). In 2002, VTEC O157:H7 was isolated from 29 (1.4%) of the 2,032 faecal samples analysed. Fourteen (48%) of the 29 strains were VTEC O157:H7 (PT4;*vtx*_2_, *vtx*_2*c*_), and these were spread over several regions other than Halland. This is highlighted in Figure 3, where the findings of VTEC O157:H7 (PT4;*vtx*_2_, *vtx*_2*c*_) in the slaughterhouse studies from 1996-97 and from 2002 are shown for comparison.

## Discussion

Between 1996 and 2002, 18 cattle farms in Sweden were implicated in transmission of EHEC infections to humans. Of these farms, 16 had cattle harbouring strains of VTEC O157:H7 belonging to phage type 4. They carried both the *vtx*_2 _and *vt*x_2*c *_genes, and clustered closely together in PFGE patterns. Strains with similar characteristics have been isolated both in a slaughterhouse and in an on-farm dairy cattle prevalence study (see below) performed in Sweden. Of the two cattle farms not having isolates of PT4, one gave VTEC O157:H7 (PT2;*vtx*_2_, *vtx*_2*c*_) isolates; the PFGE pattern of these isolates was not detected in either the slaughterhouse or the on-farm study. The second farm gave isolates of VTEC O157:H7 (PT20;*vtx*_1_, *vtx*_2*c*_) and VTEC O157:H7 (RDNC;*vtx*_1_, *vtx*_2*c*_) with two closely related PFGE patterns. The pattern of the RDNC isolate was also observed in the slaughterhouse studies, from two different slaughterhouses in central Sweden. Thus, only a limited group of genetically similar and highly pathogenic VTEC O157:H7 strains seem to be involved in direct transmission VTEC O157:H7, directly or indirectly from cattle to humans in Sweden. It has also been reported that this particular virulence pattern is predominant among strains isolated from human cases reported to the Swedish Institute of Infectious Disease Control [26] and has been the cause of two larger food-borne outbreaks of VTEC O157:H7 in Sweden [27,28]. Thus, for the period 1996-2002, the VTEC O157:H7 variant most commonly isolated from human cases in Sweden also predominated among cattle farms that were identified as the source of EHEC infections in humans. This specific variant could also be isolated from, among many others, animals sampled in the prevalence studies.

It has been reported from other countries that cattle can harbour VTEC of differing serotypes, and that direct transmission of VTEC from cattle to humans can be one cause of gastroenteritis infection (reviewed by Gyles [2]). The slaughterhouse survey on cattle faeces performed between April 1996 and August 1997 was undertaken to establish if Swedish cattle could be a source of VTEC O157:H7 for human infection. This survey showed a prevalence of 1.2% of VTEC O157:H7 isolated from Swedish cattle [19]. The prevalence of VTEC O157:H7-carrying healthy cattle in Sweden is in the range of what has been reported (0.35-15.7%) in other West European countries where extensive studies have been performed [29-35].

In contrast to the cattle strains isolated from farms associated with human infections, as described above, analyses of VTEC O157:H7 isolated from cattle sampled at slaughter showed a wide variation in variants. Likewise, when characterizing the strains isolated in the on-farm prevalence study in dairy herds, a diverse picture was seen. These strains were divided into four different known phage types, as well as some types not previously described (RDNC) and variations in verotoxin type as well as diverse PFGE patterns were observed.

Although 10 different known phage types were found among the isolates of VTEC O157 (*n *= 181) from the cattle slaughterhouse and dairy farm prevalence studies, only three types were predominant: PT4 (28%), PT8 (33%) and PT14 (18%). This dominance of a few phage types is consistent with other studies on bovine isolates [13,36,37]. When analysing strains from human cases of haemorrhagic colitis (HC) and haemolytic uraemic syndrome (HUS), the spectrum of phage types seems to be narrower [11,13] and specific phage types seem to dominate; in England and Wales, as in Finland, PT2 has often been reported in outbreak investigations [16,38]. In other countries, other phage types may predominate, e.g. in The Netherlands, Germany and Belgium, PT2 and PT4 [39], in Spain PT4 and PT8 [40] and in Scotland PT21/28 [37]. In Denmark [13] PT4 was more often associated with strains suspected of causing human illness. In Japan, however, PT32 seems to be predominant in VTEC O157 outbreaks [41]. Thus, the phage type appears not to be decisive for the pathogenic potential of VTEC O157. As more studies on strains isolated from patients include verotoxin genotyping by PCR, there is mounting evidence that the verotoxin 2 genotype of a strain is correlated both to its ability to cause infection and to the severity of the disease [4,11-16,42]. However, there are certain drawbacks when conducting such studies; often only one isolate is analysed from each patient, and it is known that infection with more than one VTEC strain can occur [16]; also, if a VTEC strain is isolated from a patient with diarrhea, no further examination need be undertaken to seek other plausible infectious agents.

The prevalence studies described here was designed to cover all of Sweden, in order to reveal any geographical differences in the occurrence of VTEC O157:H7. The bacterium was never found in bovine faecal samples collected in the north of Sweden, but was found in samples originating from all over south and central Sweden. However, most of the farms associated with human infections of VTEC O157:H7 were in the southwest. Of the 148 strains isolated in the slaughterhouse prevalence studies, 40 isolates (27%) were of PT4, verotoxin variants *vtx*_2_, *vtx*_2*c *_and had PFGE patterns identical or similar to the PT4 strains isolated from farms implicated in human cases. When mapping where VTEC O157:H7 (PT4;*vtx*_2_, *vtx*_2*c*_) strains were found, the findings in the prevalence study vis-à-vis farms connected with human cases of infection show a geographical match.

Human infections with VTEC O157:H7 have been compulsorily notifiable in Sweden since 1996. Incidence rates in Halland County during 1997 to 2002 ranged between 2.9 and 17.6 cases per 100,000 inhabitants, whereas the national Swedish average for the same period varied between 0.8 and 1.6 cases per 100,000 http://www.smittskyddsinstitutet.se. We have found that when faecal samples from cattle were collected from slaughterhouses and dairy farms all over Sweden, one specific variant, VTEC O157:H7 (PT4;*vtx*_2_, *vtx*_2*c*_) was more often found in Halland. This may explain the far higher incidence of human VTEC O157 cases in this part of Sweden. It is noteworthy that in Halland, several different VTEC O157 variants were isolated in the prevalence studies performed, but only VTEC O157:H7 (PT4;*vtx*_2_, *vtx*_2*c*_) strains were found on the 11 farms associated with human EHEC cases during the same period in that county. This finding also supports our theory that there is a particular variant of VTEC O157:H7, characterized as PT4;*vtx*_2_, *vtx*_2*c*_, that may be more pathogenic or more contagious. Despite the small number of isolates in our studies, it is worrying that in the latter prevalence studies, strains of these characteristics were found not only in Halland, but also in other parts of Sweden, which could indicate spreading of a particular pathogenic VTEC within the country.

We have found that the variants among strains of VTEC O157:H7 isolated from cattle on farms linked to human cases of infection appear to constitute a closely related cluster of isolates, in contrast to isolates collected at slaughter and in the on-farm dairy cattle prevalence study. This is consistent with the findings published by Roldgaard and colleagues[13] who showed that in Denmark the VTEC O157 strains that carry the *vtx*_2 _gene, either alone or in combination with *vtx*_2*c*_, not only cause more serious disease, but also seem more likely to be pathogenic in humans. In the present study, most of the Swedish cattle strains not associated with human infections harbour the *vtx*_2*c *_gene, either alone or combined with the *vtx*_1 _gene. Thus, the presence of the *vtx*_2 _gene, either alone or in combination with the *vtx*_2*c *_gene, could be the cause of the more aggressive behaviour of VTEC O157:H7 (PT4;*vtx*_2_, *vtx*_2*c*_). Other factors, such as other specific virulence factors, acid tolerance and environmental survival, not investigated in this study, may of course be of importance.

## Conclusions

We have found that in Sweden isolates of VTEC O157:H7 strains characterized as being PT4; *vtx*_2_;*vtx*_2*c *_were the cause of most cattle-to-human transmitted EHEC cases connected with farm visits between 1996 and 2002. We speculate that the reason for this could be that this particular variant of VTEC O157:H7 is more pathogenic or more contagious than other variants found in the Swedish cattle population. Further studies on this particular variant of VTEC O157:H7 regarding farm epidemiology, toxin production and other virulence factors are therefore warranted.

## Authors' contributions

Both authors (AA and EE) outlined the design, coordinated the study and drafted the manuscript. AA coordinated and evaluated the molecular analysis. Both authors read and approved the final manuscript.
